# Ultrasonography of the nuchal translucency of healthy and abnormal English Bulldog fetuses

**DOI:** 10.1590/1984-3143-AR2021-0023

**Published:** 2021-12-10

**Authors:** Letícia Pavan, Beatriz Gasser, Marjury Cristina Maronezi, Igor Cezar Kniphoff da Cruz, Rafael Kretzer Carneiro, Priscila Silva, Ricardo Andrés Ramirez Uscategui, Luciana Cristina Padilha-Nakaghi, Marcus Antônio Rossi Feliciano

**Affiliations:** 1 Departamento de Reprodução Animal, Universidade Estadual Paulista, Jaboticabal, SP, Brasil; 2 Universidade Federal do Vales do Jequitinhonha e Mucuri, Unaí, MG, Brasil; 3 Departamento de Clínica de Grandes Animais, Universidade Federal de Santa Maria, Santa Maria, RS, Brasil

**Keywords:** anasarca, canine, nuchal edema, nuchal translucency, ultrasound

## Abstract

The aim of this study was to measure the nuchal translucency (NT) of canine fetuses to establish reference values ​​for healthy gestational processes and to verify its effectiveness in the diagnosis of congenital abnormalities. On day 34 of gestation, the NT of three fetuses from each of the 26 English bulldog female dogs was measured. The first fetus was the one located immediately cranial to the bladder, the second was selected from the left side of the abdomen, and the third from the right side. The reference values for healthy animals were offset using descriptive statistics. The diagnostic ability of the test to identify fetal malformations was studied using receiver operating characteristic curve analysis. Of the 26 litters, only 18 had healthy fetuses, 4 had fetuses with anasarca, 3 had fetuses with abdominal wall defects, and 1 had both types of abnormalities. The NT was higher in canine fetuses that presented anasarca in the litter than in normal litters (1.8 ± 0.77 mm vs. 1.4 ± 0.48 mm; P = 0.0249), with a cut-off value of NT > 1.45 mm (sensitivity = 61.54%, specificity = 70.18%). NT greater than 1.45 mm seems to be a diagnostic tool for the identification of anasarca during gestation of bulldogs. Considering the unprecedented use of this parameter in canine species and the limitations found during the study, further studies will be needed in order to use it on clinical practice.

## Introduction

Measurement of nuchal translucency (NT) is part of the ultrasound examination in the prenatal care of women. This measurement comprises the maximum thickness of the subcutaneous translucency between the skin and the soft tissue of the cervical spine (Nicolaides et al., 1992a). It is obtained in a medium sagittal section image of the fetus, in which a thicker region of translucency must be selected, to distinguish the fetal skin from the amnion. In humans, the ideal time for this evaluation is between the 11^th^ and 14^th^ gestational weeks (Kline-Fath et al., 2015).

The increase in NT thickness was initially related to chromosomal abnormalities, mainly in human trisomy 21. NT measurement is an important tool for deciding to perform invasive karyotyping tests by amniocentesis and/or chorion villus sampling (Nicolaides et al., 1994). Other abnormalities such as trisomy 13 and 18, as well as triploidy, were also associated with nuchal edema in this species (Nicolaides et al., 1992b).

Subsequently, it was found that even in fetuses with normal karyotype, increased NT is significant as an effective screening for defects of the heart, large vessels, and skeletal dysplasia; increased NT is also related to higher abortion rates and perinatal death (Nicolaides et al., 2002). As the NT thickness increases, several structural defects and the prevalence of genetic syndromes such as severe heart defects, diaphragmatic hernia, and omphalocele increase (Souka et al., 1998). In humans, different reference values have been used over the years, and although controversy remains, there is a “consensus” that NT greater than 3.5 mm is elevated (Bakker et al., 2014).

It is believed that the pathophysiology of nuchal edema is multifactorial because it is related to numerous anomalies. One factor could be cardiac dysfunction, since there is a strong association between abnormalities of the heart and large arteries with the increase in NT, both in fetuses with normal and abnormal karyotypes. Other factors include venous congestion in the head and neck (due to constriction of the fetal body), failure in lymphatic drainage (due to abnormal development of the lymphatic system or fetal movements impaired by neuromuscular disorders), the altered composition of the extracellular matrix (as this condition is present in several genetic syndromes), anemia, and fetal hypoproteinemia (Souka et al., 2005).

To the best of our knowledge, there are no reports of NT ultrasound assessment in dogs. The aim of this study was to measure the NT of canine fetuses, to establish reference values for healthy bulldogs and to verify if this parameter can help in the early diagnosis of fetal abnormalities in this species.

## Material and methods

The Animal Ethics and Welfare Committee of the institution approved this study (protocol no. 008364/18). A prospective study was conducted with animals from a commercial kennel, whose owner signed a free consent form after being fully informed about the study.

Twenty-six English bulldog bitches, aged between 1 and 4.5 years were used, after confirming their health status by anamnesis (history of reproductive health), complete blood cell count, and physical and obstetric examinations (inspection of the reproductive tract).

From the beginning of the proestrus (determined by the presence of vulvar edema and serosanguinous vaginal discharge), vaginal cytology of the female dogs was performed every 48 h, until the presence of > 90% of cornified epithelial cells which indicated the onset of estrus (Kutzler et al., 2003). During estrus, serum progesterone concentration (P4) was measured every 48 hours by the chemiluminescence method (IMMULITE®1000 Progesterone – Siemens®) and the time of ovulation was considered at the first day in which P4 > 4.0 mg/ml was observed (Gloria et al., 2018). The females were subjected to vaginal artificial insemination (AI) with fresh semen, starting from the time of ovulation, in a protocol of three inseminations, with an interval of 48 hours between each one. The day of the first AI was considered the first day of gestation.

For ultrasound examinations, hair clipping of the female abdomen was performed between the xiphoid appendix and the last two pairs of mammary glands, extending laterally in the ventral region to the lumbar muscles, close to the last pair of ribs. Through manual limb restraint, female dogs were placed in dorsal-lateral recumbency, with the head toward the monitor and the body parallel to the equipment. Before the examination, a specific gel was applied.

Ultrasound examinations were performed using the ACUSON S2000® (Siemens®, Munich, Germany) ultrasound system equipped with a 9.0 MHz linear transducer. The 34^th^ gestational day was defined as the ideal time for the visualization and measurement of NT, and the maximum NT thickness was observed in our previous research that evaluated the bitches daily. The evaluation on the 34^th^ gestational day was determined in the pre-experiment and correlated with the evaluation period in human medicine, i.e., 10-14 weeks in humans (Hyett et al., 1999).

Ultrasonography was performed in the pelvic region, proceeded to the left abdominal region, and then to the right abdominal region, aiming to examine and differentiate all fetuses. The image adjustments (focus, gain, and depth) were adequate for each fetus, with a maximum depth of 4 cm (Simões et al., 2018).

After locating the nuchal region of the fetus, NT was measured as the thickness between the skin and soft tissues of the neck. This procedure was repeated in three different fetuses of each female. The first was the one located immediately cranial to the bladder, the second and the third were selected from the most caudal region of the left and the right side of the abdomen, respectively. When the pregnancies were of one or two concepts, all fetuses were evaluated. The maximum number of three fetuses was determined to prevent the exam delay causing stress in pregnant bitches. An ultrasound examination was also performed at 49 and 60 days of gestation to monitor the development of pregnancy.

To ensure an adequate measurement of the NT, it was necessary to differentiate the skin from the amniotic membrane by sagittal section scanning by moving the transducer to the right and left sides of the fetus. Then, an image of the fetus was taken in a medium sagittal section, and some fetuses had the abdomen and others had the back in the proximal region of the image, depending on their position in the maternal uterus. When this image was properly obtained, NT was measured in the cervical region at the site with the largest thickness, by placing the calipers from the skin to the soft tissue covering the cervical spine, providing measurements in millimeters.

The day of parturition was estimated by ovulation day and clinical signs of parturition proximity (nesting behavior, rectal temperature decline, and vulvar discharge). All dogs underwent cesarean section, and the procedure was indicated when the heart rate of at least one of the fetuses considered healthy remained between 160 and 180 bpm (Gil et al., 2014).

Statistical analysis was performed using R® software (R Foundation for Statistical Computing, Vienna, Austria). The normality (Shapiro's test) and homoscedasticity (Bartlett's test) of the NT were tested. Descriptive statistics were calculated for animals with normal litters to establish reference parameters. Subsequently, the NT of fetuses from normal litters and litters with neonatal malformations were compared by t-Student test and those that were significant were subjected to discriminative power analysis (normal litters vs. litters with fetal malformations) through receiver operating characteristic curves (ROC) and cut-off value, sensitivity, specificity, likelihood ratio, area under the curve (AUC), positive predictive value (PPV), negative predictive value (NPV), and accuracy were calculated using the logistic regression model in order to identify the diagnostic ability of fetal malformations through NT; the significance was set at P < 0.05 for all tests and the results are expressed as mean ± standard deviation (SD).

## Results

The 26 bitches evaluated were 29 ± 10 (12-54) months old and weighed 24.4 ± 2.4 (18.8-32.2) kilograms. Twelve (46.15%) were primiparous. The mean gestation length was 62.3 ± 1.4 (59-66) days, the litter size was 5.6 ± 2.1 (2-12) puppies, and the total number of puppies was 147 (one of them stillborn, which had no abnormalities).

In each female, the NT of three fetuses was measured, except for three bitches that had only two fetuses. Therefore, 75 fetuses were evaluated. Of the 26 litters studied, 18 resulted in only healthy neonates, four resulted in fetuses with anasarca, three resulted in fetuses with abdominal wall defects, and one resulted in both types of neonatal abnormalities. Considering the 147 neonates, eight (5.44%) showed signs of anasarca and seven (4.76%) had abdominal wall defects.

**Figure 1:**
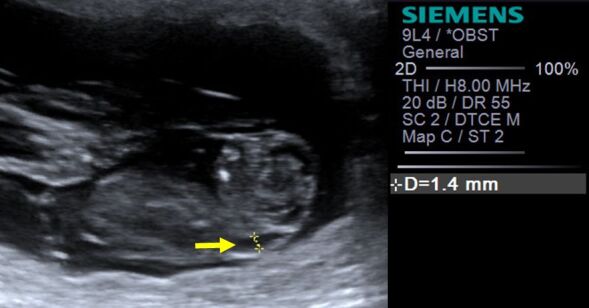
An ultrasonography image of NT (nuchal translucency) measurement (arrow) in a healthy canine fetus (NT = 1.4mm), at 34 days of gestation.

On the 34^th^ gestational day, it was possible to detect in the ultrasound examination the presence of a translucent line between the skin and soft tissues above the cervical spine, corresponding to NT (Figure 1). All NT were anechoic and their measurements ranged from 0.8 to 3.5 mm.

**Figure 2:**
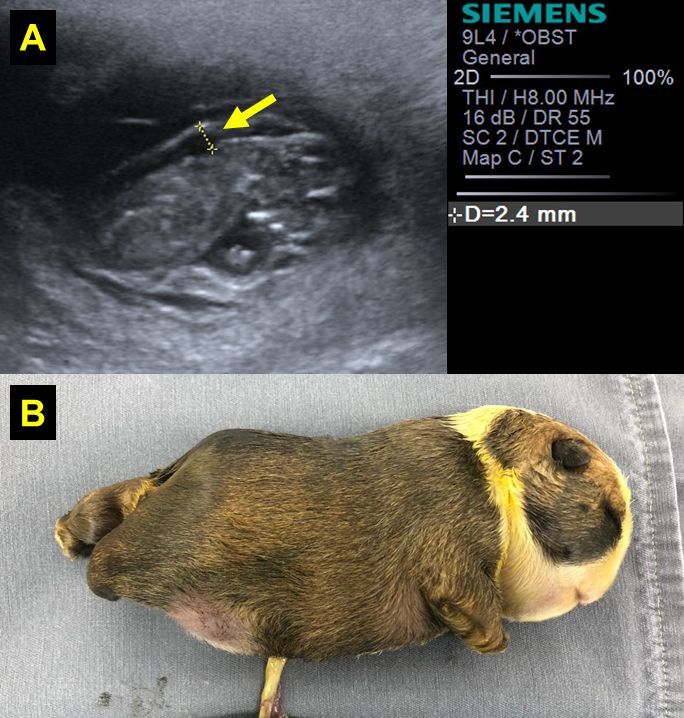
(A) An ultrasonography image of increased NT (nuchal translucency - arrow) measurement (NT = 2.4mm) in a canine fetus, at 34 days of gestation; (B) Fetus of image A, exhibiting anasarca at birth.

NT resulted higher (P = 0.0249) in the fetuses of the bitches that gave birth to neonates with anasarca (1.8 ± 0.8 mm) (Figure 2) than in fetuses of litters without apparent malformations (1.4 ± 0.5 mm). In turn, in fetuses born with abdominal wall defects, NT (1.5 ± 0.7 mm) resulted similar (P = 0.8359) to that found in fetuses of normal litters (Table 1).

**Table 1:** Descriptive statistics for nuchal translucency (NT) evaluated in canine fetuses at 34^th^ day of gestation in 26 bulldog bitches, of which, 18 gave birth only to healthy neonates, 4 fetuses with anasarca, 3 fetuses with abdominal wall defects and 1 both types of fetal abnormalities.

**Descriptive statistic**	**Litters of healthy fetuses**	**Litters with Anasarca**	**Litters with abdominal wall defects**
Number of fetuses evaluated	60	8	7
Minimum	0.80	1.00	1.00
Maximum	3.30	3.50	3.50
Mean	1.42	1.81	1.51
Std. Deviation	0.49	0.78	0.68
Std. Error of Mean	0.06	0.22	0.19
Lower 95% CI of mean	1.29	1.34	1.08
Upper 95% CI of mean	1.55	2.28	1.93
Coefficient of variation (%)	34.18	43.00	44.38

Std: deviation; CI: confiance intervable.

**Figure 3:**
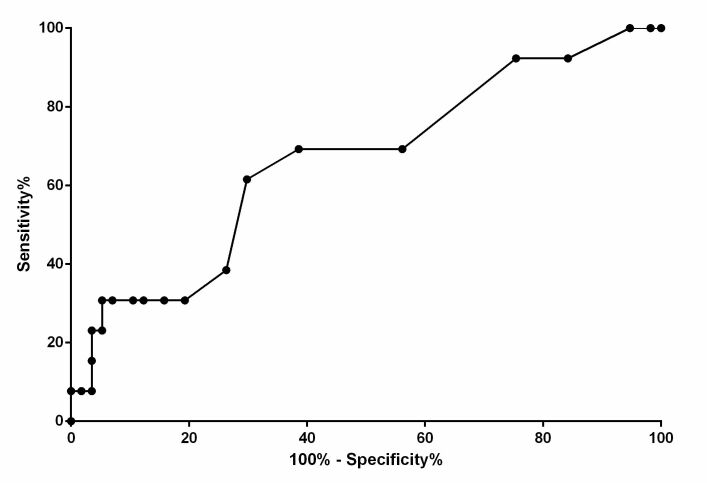
Receiving operating characteristic (ROC) curve of discriminative power analysis (normal litters vs. litters with anasarca) for nuchal translucency (NT) evaluated in canine fetuses at 34^th^ day of gestation in 26 English bulldog bitches, of which, 18 resulted in healthy neonates and 4 resulted in fetuses with anasarca, diagnostic sensitivity and specificity are reported.

According to the ROC analysis, an NT greater than 1.45 is indicative of fetuses with anasarca with a sensitivity of 61.54%, specificity of 70.18%, the likelihood ratio of 2.063, AUC of 66.13%, PPV of 32%, NPV of 88%, and accuracy of 69%. The prevalence of anasarca in this study was 18.6% (Figure 3).

There were no ultrasound signs of malformation at 49 days of gestation in two of the five bitches that gave birth to neonates with anasarca; however, at 60 days, it was observed that one or more fetuses developed ultrasound signals of such alteration.

## Discussion

This study showed that it is possible to recognize nuchal edema in canine fetuses by sonographic examination at 34 days of gestation in canine females. According to the results obtained, female dogs whose fetuses had NT greater than 1.45 mm were twice as likely to deliver fetuses with anasarca. Similarly, the measurement of NT in humans is an important diagnostic tool, since an increase in its thickness has a strong association with chromosomal defects and malformations in chromosomally normal fetuses (Nicolaides et al., 1992a; Souka et al., 2005). There are no reports on the measurement of NT in species other than humans.

Anasarca, which in many cases is of unknown cause in the canine species, consists of subcutaneous edema that may or may not be accompanied by peritoneal, pleural, and pericardial effusion. It is a congenital abnormality frequently described in English Bulldogs, which predisposes to dystocia and has high neonatal mortality (Hopper et al., 2004; Cunto et al., 2015). Thus, diagnosis during pregnancy may be an important factor for planning cesarean sections or establishing therapeutic measures if an intrauterine therapeutic alternative is developed for this condition. In this sense, NT presents itself as a promising tool for the diagnosis of this fetal alteration with moderate sensitivity and specificity and acceptable NPV. Although a reduced PPV was observed, it might have resulted as a consequence of not having evaluated and identified all fetuses of the litter and thus, accurately recognized sick fetuses, which is a limitation of this study.

Heart defects are the main cause of increased NT in human euploid fetuses (Souka et al., 1998) and the prevalence of this type of abnormality increases with the thickness of NT, being 2.89%, 9.09%, and 19.51% when the NT was 3.5 to 4.4 mm, 4.5 to 5.4 mm, and greater than 5.4 mm, respectively (Hyett et al., 1999). Countless cardiac lesions have already been identified in bulldog fetuses with anasarca, the most frequent being ventricular septal defects, abnormal heart shapes, double apices, and micro-hearts (Padgett et al., 1986). Of the 22 fetuses analyzed by these latter authors, only four had a normal heart, and most of them had more than one heart defect. In the present study, cardiac abnormalities were not examined or detected in fetuses with anasarca, because the use of fetal echocardiography is still recent in dogs (Giannico et al., 2016), we don't have the preparation for this exam and no necroscopy was performed. Therefore, it is not possible to state that cardiac defects are associated with the etiology that promotes increased NT in canine fetuses, as it is in humans.

The prevalence of omphalocele, as well as severe heart defects, was substantially higher in 4116 human pregnancies with increased NT and chromosomally normal fetuses (Souka et al., 1998). However, in the present study, NT showed no relationship with abdominal wall defects. As NT measurements were made in only three fetuses per litter, selected according to their position in the abdomen of the bitch, there is a possibility that the fetuses with omphalocele were not selected, and this could be a limitation of this study.

In human fetuses examined between the 10^th^ and 14^th^ gestational weeks, a PPV of 6.3% and an NPV of 99.9% was determined for the main congenital heart defects when the NT was greater than 3.5 mm (Hyett et al., 1999), following a pattern similar to the results found in our study for anasarca identification in dog fetuses. Consequently, in our study, this measure proved to be a good exclusion parameter for anasarca at the 34^th^ day of gestation, since the probability of a canine pregnancy not resulting in fetuses with such alteration (NPV) was 88%, when NT was less than 1.45 mm. However, it is not a parameter that effectively identifies this change in all cases because the PPV was 32%.

It was possible to establish the ideal moment for measuring NT in female dogs by comparing the embryonic development of humans and dogs. At the end of the first gestational trimester in women, organogenesis is complete at the 13^th^ post-menstrual week (Woodward, 2016). In the canine species, this event ends later, in the second trimester, at 35 days of gestation (Pretzer, 2008). Thus, the 34^th^ gestational day, which is the final moment of organogenesis is the best moment determined in this study to make the measurement, resembling the best period in humans to perform the examination.

As it is a multiparous species, it is guaranteed that in healthy litters, only fetuses without abnormalities were evaluated. However, in litters with malformations, it is possible that healthy fetuses were randomly selected for data collection, which is recognized as a principal limitation of this study and ultrasound technique.

Considering that our experimental model was a specific breed, the English bulldog, the described technique should be applied to other breeds to check if they have the same TN values for healthy fetuses. In addition to standardization for each specific breed, we recommend that studies be carried out to effectively identify fetuses with abnormalities so that it is possible to compare and establish accurate diagnostic data for each patient.

## Conclusions

NT can be measured in English bulldog fetuses at 34 days of gestation, with reference values between 1.29 and 1.55 mm. This parameter seems to be a diagnostic tool for the prediction of anasarca in bulldog fetuses, with a cutoff greater than 1.45 mm, however, this diagnostic tool should be used with caution, since it presents moderate sensitivity and specificity results. In addition, more studies with this and other breeds in association with a larger sample are treated to standardize the technique, should be needed in order to use this parameter in clinical practice.
